# Social Service at St. Thomas's Hospital

**Published:** 1918-08-17

**Authors:** A. E. Cummins

**Affiliations:** Lady Almoner. St. Thomas's Hospital, Lady Almoner's Office, London, S.E.


### Social Service at St. Thomas's.

*To the Editor of* The Hospital.

Sir,—While acknowledging the generous appreciation of the Social Service Department at St. Thomas's Hospital in your issue of August 3 I should like to draw your attention to one point—namely, a paragraph in which you refer in somewhat damaging terms to the work of the Charity Organisation Society. As St. Thomas's Hospital has been indebted to the Charity Organisation Society for at least fourteen years for an immense amount of help, both in inquiry work and also in material relief for patients, it would be hardly excusable did I allow your statement to pass without comment. In addition to this I personally owe a deep debt of gratitude to Sir Charles Loch, Mr. Henry Toynbee, and many other members of the Charity Organisation Society for their help and inspiration in my work here. I also have to thank the Charity Organisation Society for a long course of training in the scientific methods of relief.—Yours truly,

A. E. Cummins, Lady Almoner.

St. Thomas's Hospital, Lady Almoner's Office,
London, S.E., August 2, 1918.

